# Improving the Quality and Completeness of Cesarean Section Operative Note Documentation Through a Structured Proforma: A Closed-Loop Clinical Audit

**DOI:** 10.7759/cureus.113968

**Published:** 2026-08-04

**Authors:** Abdalmahmoud Asadig Kanan Ahmed, Ola Muawia Ali Ahmed, Marina Hageb Kamal Hageb, Mozn Alfadil Abdelmahmoud Ahmed, Mortada Abu Sabah Elsheikh Mohamed Ahmed, Howida Hassan, Fatima Mohamed Hassan Mohd Elhassan, Sara Suliman, Rehab Hassan Abdelmajeed Musned, Roua Musadag Ali M Elhassan, Gehan Mohammed Hamad Mohammed, Khadeja Fadlallah Abdallah Ebrahim, Amna G Salih, Mawada Faisal Abdelgalil Nugud, Rashad El-Maddah Mohamed Mostafa Gamea, Soha Mahmoud Abdorhman Minees, Rimaz Maccido Ibrahem Ahmad, Mustafa Mohamed

**Affiliations:** 1 General Surgery, Al-Managil Teaching Hospital, Al-Managil, SDN; 2 Obstetrics and Gynecology, Al-Managil Teaching Hospital, Al-Managil, SDN; 3 Internal Medicine, University of Medical Sciences and Technology (UMST), Khartoum, SDN; 4 Obstetrics and Gynecology, King Salman Hospital, Riyadh, SAU; 5 Obstetrics and Gynecology, Noonu Atoll Hospital, Manadhoo Island, MDV; 6 Internal Medicine, Al Neelain University, Khartoum, SDN

**Keywords:** cesarean section, clinical audit, clinical governance, obstetric surgery, operative documentation, operative note proforma, patient safety, quality improvement, sudan

## Abstract

Background

High-quality operative documentation is essential for patient safety, continuity of care, clinical governance, and medicolegal accountability. Despite established professional guidance, deficiencies in cesarean section (CS) operative note documentation remain common in many healthcare settings. This audit aimed to determine the effect of implementing a structured operative note proforma, supported by educational sessions, departmental feedback, and visual reminder posters, on compliance with operative documentation standards.

Methods

A closed-loop clinical audit was conducted at Al-Managil Teaching Hospital, Sudan. The first audit cycle was conducted between 20/10/2025 and 19/11/2025, during which 80 consecutive CS operative notes were reviewed. Baseline assessment evaluated compliance with predefined Royal College of Obstetricians and Gynaecologists (RCOG) documentation standards, including mandatory medicolegal documentation, clinical operative details, neonatal information, and postoperative management. Following the baseline assessment, a multifaceted intervention was implemented between 20/11/2025 and 19/01/2026, including the introduction of a structured operative note proforma, educational sessions, departmental feedback meetings, and visual reminder posters. The second audit cycle was conducted between 20/01/2026 and 19/04/2026, during which 120 consecutive operative notes were evaluated using identical audit criteria. Compliance rates between audit cycles were compared using Pearson's chi-square test.

Results

Baseline compliance with documentation standards was suboptimal across mandatory documentation, clinical operative details, and postoperative management domains. Following implementation of the intervention package, significant improvements were observed across all assessed parameters. Documentation of patient identifiers, operative details, neonatal information, and postoperative management plans improved substantially, with most indicators achieving complete or near-complete compliance during the second audit cycle. All improvements were statistically significant (p < 0.001).

Conclusions

Implementation of a structured operative note proforma combined with targeted educational and feedback interventions significantly improved the quality and completeness of CS operative documentation. Structured quality improvement approaches represent an effective strategy for enhancing documentation standards and strengthening clinical governance within obstetric practice.

## Introduction

Cesarean section (CS) is one of the most commonly performed major surgical procedures worldwide and remains an essential intervention for reducing maternal and neonatal morbidity and mortality when clinically indicated. Over recent decades, the global rate of cesarean delivery has increased substantially, making the quality of perioperative care and documentation an increasingly important component of obstetric practice. Although CS is frequently lifesaving, it is associated with significant intraoperative and postoperative risks, including hemorrhage, infection, thromboembolic complications, anesthetic-related events, and complications affecting future pregnancies. Consequently, comprehensive operative documentation is critical to ensure safe clinical management and continuity of care for both mother and newborn [1].

Operative notes constitute a fundamental medicolegal and clinical record of surgical care. They provide detailed information regarding the indication for surgery, operative findings, surgical techniques performed, personnel involved, intraoperative complications, estimated blood loss, neonatal outcomes, and postoperative management plans. Accurate documentation facilitates communication among multidisciplinary healthcare teams and supports clinical decision-making during postoperative follow-up. In contrast, incomplete or inaccurate operative records may compromise patient safety, impair continuity of care, hinder future obstetric planning, and increase medicolegal vulnerability for healthcare providers and institutions [2,3].

Professional organizations including the Royal College of Obstetricians and Gynaecologists (RCOG) and the Royal College of Surgeons (RCS) emphasize that operative documentation should be clear, contemporaneous, comprehensive, and standardized. International guidance recommends that operative notes be completed immediately after surgery and include all essential clinical documentation required to accurately describe the procedure performed, guide subsequent patient management, support professional accountability, and provide a contemporaneous record that may be used in medicolegal proceedings when necessary [4,5].

Despite the availability of established documentation standards, numerous studies have demonstrated persistent deficiencies in operative note quality across different surgical specialties, including obstetrics and gynecology. Audits of CS documentation have reported frequent omissions involving operative findings, identification of surgical personnel, estimated blood loss, postoperative instructions, neonatal information, and documentation of complications. These deficiencies indicate substantial variation between recommended standards and actual clinical practice and highlight the need for structured quality improvement initiatives aimed at enhancing documentation completeness and accuracy [2,6].

Clinical audit is a recognized quality improvement methodology that systematically evaluates current practice against predefined standards and promotes evidence-based change through implementation of targeted interventions and subsequent reassessment. In surgical and obstetric practice, regular auditing of operative documentation plays a crucial role in identifying deficiencies, improving professional accountability, strengthening patient safety measures, and ensuring adherence to accepted clinical standards. Previous quality improvement studies have demonstrated that interventions such as structured operative note templates, staff education, visual reminders, and continuous feedback can significantly improve documentation quality and compliance with professional guidelines [3,5].

At Al-Managil Teaching Hospital, the quality and completeness of CS operative note documentation had not previously been comprehensively evaluated against internationally recognized standards. Given the importance of accurate operative records for patient safety, professional accountability, postoperative management, future obstetric care, and continuous quality improvement, this closed-loop clinical audit was undertaken to determine the effect of implementing a structured operative note proforma, supported by educational sessions, departmental feedback, and visual reminder posters, on compliance with the RCOG operative documentation standards through a two-cycle clinical audit.

## Materials and methods

This study was conducted as a closed-loop clinical audit and quality improvement project at Al-Managil Teaching Hospital, a tertiary referral hospital in Gezira State, Sudan. The project aimed to evaluate and improve the quality and completeness of CS operative note documentation in accordance with internationally recognized standards for operative recordkeeping. The audit followed the standard clinical audit cycle consisting of baseline assessment, implementation of targeted quality improvement interventions, and reaudit to evaluate the effectiveness of the implemented changes.

The audit standard was derived from the RCOG Good Practice Guidance on Operative Documentation, which outlines the essential documentation elements required for clinical care, professional accountability, operative details, neonatal information, and postoperative management that should be recorded in every CS operative note. In accordance with the RCOG recommendations, the audit aimed to achieve complete documentation of all mandatory documentation elements. A target compliance rate of 100% was set for mandatory documentation items, while a pragmatic benchmark of at least 95% was adopted for the remaining clinical, operative, neonatal, and postoperative documentation domains, reflecting an achievable quality improvement target rather than a difference in their clinical importance.

The first audit cycle was conducted over one month between October 20, 2025, and November 19, 2025. During this phase, operative notes of women who underwent CS at Al-Managil Teaching Hospital were reviewed retrospectively to determine baseline compliance with the predefined audit criteria. A total coverage (consecutive sampling) approach was adopted, whereby all eligible consecutive CS operative notes generated during each audit period were included. This resulted in the inclusion of 80 operative notes in the first audit cycle.

Following completion of the baseline assessment, an intervention phase was undertaken over two months from November 20, 2025, to January 19, 2026. The intervention strategy was multifaceted and designed to address the deficiencies identified during the first audit cycle. Educational sessions were conducted for obstetric medical officers, registrars, specialists, and theatre staff to reinforce the importance of comprehensive operative documentation and to familiarize clinicians with the RCOG documentation standards. Departmental meetings were organized to discuss baseline audit findings, highlight areas requiring improvement, and encourage collective responsibility for documentation quality. Additionally, educational posters summarizing the essential components of CS operative notes were displayed in operating theatres, labor wards, and doctors’ offices as visual reminders. To facilitate standardized documentation, a structured operative note template based on RCOG recommendations was developed, printed, and introduced into routine clinical practice. The template was designed to ensure systematic recording of mandatory patient identifiers, operative findings, neonatal information, and postoperative management plans while minimizing omissions and variability in documentation.

The second audit cycle was conducted prospectively over a three-month period from January 20, 2026, to April 19, 2026. Using the same methodology and audit criteria employed in the first cycle, all consecutive CS operative notes completed during the reaudit period were assessed. A total of 120 operative notes were included in the second audit cycle. This allowed direct comparison of compliance rates before and after implementation of the quality improvement interventions.

The audit included all women who underwent CS at Al-Managil Teaching Hospital during the specified audit periods and whose handwritten paper operative notes were available for review. At the time of the audit, operative documentation was completed using handwritten paper records as part of routine clinical practice. Cases were excluded if operative notes were missing from the patient file, were illegible to the extent that assessment of documentation quality was not possible, or lacked sufficient information for evaluation against the audit criteria. No operative notes met the exclusion criteria during either audit cycle.

Data collection was performed using a structured audit checklist developed directly from the RCOG Good Practice Guidance on operative documentation. The checklist assessed multiple domains of operative note quality, including patient demographics, obstetric characteristics, operative personnel, intraoperative findings, surgical details, neonatal information, and postoperative management plans. The complete structured audit checklist used for data collection is provided in (Appendix 1). Data extraction was carried out by five members of the audit team who underwent a standardized orientation session before data collection. The orientation included review of the audit objectives, RCOG documentation standards, operational definitions for each audit variable, and standardized use of the structured audit checklist to promote consistency in data extraction. To ensure consistency in data collection, all reviewers used the same standardized data collection tool and predefined operational definitions for each audit variable.

The primary outcome measure was the proportion of operative notes that complied with each documentation standard. Secondary outcome measures included overall improvement in documentation quality following implementation of the intervention package and achievement of the predefined audit targets. Compliance rates were calculated for each documentation item and compared between audit cycles.

Data were entered into MS Excel (Microsoft Corporation, Redmond, Washington, United States) and subsequently analyzed using JASP statistical software version 0.96.0.0 (Released 2026; University of Amsterdam, Amsterdam, The Netherlands). Descriptive statistics were used to summarize compliance rates as frequencies and percentages. Inferential statistical analysis was performed using the Pearson chi-square test to compare compliance rates between the first and second audit cycles. Statistical significance was defined as a two-sided p-value of less than 0.05.

The project was conducted as a quality improvement initiative aimed at enhancing documentation standards and patient care within the obstetrics and gynecology department. Ethical approval was obtained from the Institutional Review Board of Al-Managil Teaching Hospital prior to commencement of the audit (Ref. No. 2026-AL-055). All collected data were anonymized prior to analysis, and patient confidentiality was maintained throughout the study. No patient identifiers were recorded during data collection, and no modifications were made to routine patient management as part of the audit process. As this project constituted a clinical audit and quality improvement initiative utilizing routinely collected healthcare data without direct patient intervention, the requirement for individual informed consent was waived by the Institutional Review Board. The project was conducted in accordance with the principles of clinical governance, quality improvement, and the ethical principles outlined in the Declaration of Helsinki.

## Results

During the study period, all consecutive CS operative notes generated during both audit cycles were screened for eligibility. No operative notes were missing, excluded, or deemed ineligible due to illegibility or incomplete records. Consequently, all 80 operative notes from the baseline audit cycle and all 120 operative notes from the reaudit cycle were included in the final analysis, resulting in a total of 200 operative notes evaluated (Table 1).

**Table 1:** Compliance with mandatory documentation standards before and after implementation of the quality improvement intervention Compliance with mandatory documentation standards during the baseline audit cycle (October 20-November 19, 2025) and the reaudit cycle (January 20-April 19, 2026) following the implementation of targeted quality improvement interventions. Comparisons between audit cycles were performed using Pearson's chi-square test. A p-value of <0.05 was considered statistically significant

Documentation item	Cycle 1 (n = 80) n (%)	Cycle 2 (n = 120) n (%)	χ²	p-value
Patient name documented	30 (37.5)	120 (100.0)	96.69	<0.001
Patient age documented	3 (3.8)	116 (96.7)	168.14	<0.001
Date documented	34 (42.5)	118 (98.3)	79.00	<0.001
Indication for cesarean section documented	34 (42.5)	120 (100.0)	86.39	<0.001
Primary surgeon documented	34 (42.5)	120 (100.0)	86.39	<0.001
Type of anesthesia documented	34 (42.5)	120 (100.0)	86.39	<0.001
Time of delivery documented	27 (33.8)	120 (100.0)	104.79	<0.001
Clinician name documented	5 (6.3)	120 (100.0)	176.02	<0.001
Date and time of documentation recorded	12 (15.0)	120 (100.0)	150.78	<0.001
Signature documented	5 (6.3)	120 (100.0)	176.02	<0.001

Baseline compliance with mandatory documentation standards was generally poor, with documentation rates ranging from 3.8% for patient age and 6.3% for clinician name and signature to 42.5% for date of operation, indication for CS, primary surgeon identification, and type of anesthesia. Documentation of time of delivery was recorded in only 33.8% of operative notes, while documentation of the date and time of note completion was present in 15.0% of records.

Following the introduction of the structured operative note proforma, educational sessions, departmental meetings, and visual reminder posters, marked improvements were observed across all mandatory documentation domains. Compliance reached 100% for patient name, indication for CS, primary surgeon identification, type of anesthesia, time of delivery, clinician name, documentation signature, and date and time of documentation. Documentation of patient age and date of operation also improved significantly, reaching 96.7% and 98.3%, respectively.

All mandatory documentation items demonstrated statistically significant improvements between audit cycles (p < 0.001 for all comparisons). The greatest improvements were observed in clinician identification and signature documentation, both of which increased from 6.3% at baseline to 100% following intervention (χ² = 176.02; p < 0.001). Similarly, documentation of patient age improved from 3.8% to 96.7% (χ² = 168.14; p < 0.001), while recording of the date and time of documentation increased from 15.0% to 100% (χ² = 150.78; p < 0.001).

A marked improvement was observed across all clinical operative documentation domains following the implementation of the quality improvement interventions (Table 2). Baseline compliance was particularly poor for documentation of gestational age, gravidity and parity, assistant surgeon identification, membrane status, and liquor status, with compliance rates ranging from 0.0% to 2.5%. Similarly, documentation of neonatal parameters, including Apgar score, birth weight, and newborn condition, was inconsistently recorded during the first audit cycle.

**Table 2:** Compliance with clinical operative documentation standards Compliance with clinical operative documentation standards during the baseline audit cycle (October 20-November 19, 2025) and the reaudit cycle (January 20-April 19, 2026) following the implementation of the quality improvement intervention package. Comparisons were performed using Pearson's chi-square test. A p-value of <0.05 was considered statistically significant

Documentation item	Cycle 1 (n = 80) n (%)	Cycle 2 (n = 120) n (%)	χ²	p-value
Gravidity and parity documented	1 (1.3)	118 (98.3)	190.08	<0.001
Gestational age documented	0 (0.0)	116 (96.7)	185.47	<0.001
Assistant surgeon documented	2 (2.5)	120 (100.0)	190.17	<0.001
Anesthetist documented	15 (18.8)	120 (100.0)	138.54	<0.001
Type of abdominal incision documented	34 (42.5)	120 (100.0)	86.39	<0.001
Type of uterine incision documented	8 (10.0)	120 (100.0)	171.43	<0.001
Membrane status documented	2 (2.5)	120 (100.0)	190.17	<0.001
Liquor status documented	2 (2.5)	120 (100.0)	190.17	<0.001
Intraoperative findings documented	30 (37.5)	120 (100.0)	96.69	<0.001
Procedure details documented	38 (47.5)	120 (100.0)	74.44	<0.001
Estimated blood loss documented	26 (32.5)	120 (100.0)	107.44	<0.001
Intraoperative complications documented	14 (17.5)	118 (98.3)	147.29	<0.001
Apgar score documented	12 (15.0)	120 (100.0)	150.78	<0.001
Birth weight documented	15 (18.8)	109 (90.8)	106.62	<0.001
Baby condition at birth documented	24 (30.0)	118 (98.3)	108.11	<0.001

Following the introduction of the structured operative note template and targeted educational interventions, substantial improvements were observed across all assessed domains. Compliance reached 100% for assistant surgeon identification, anesthetist documentation, abdominal and uterine incision types, membrane status, liquor status, intraoperative findings, procedure details, estimated blood loss, and Apgar score documentation. Documentation of gestational age and gravidity/parity increased to 96.7% and 98.3%, respectively, while birth weight documentation improved from 18.8% to 90.8%. Documentation of intraoperative complications and neonatal condition increased to 98.3%.

All assessed clinical operative documentation standards demonstrated statistically significant improvement between audit cycles (p < 0.001 for all comparisons), indicating a substantial positive impact of the implemented quality improvement measures on the completeness of CS operative records.

Documentation of postoperative management plans improved significantly following the implementation of the quality improvement interventions (Table 3). During the baseline audit cycle, compliance with postoperative documentation standards was generally poor, ranging from 2.5% for wound care instructions and 8.8% for postoperative vital signs to 46.3% for antibiotic prescription documentation. Documentation of feeding plans and follow-up arrangements was also inconsistent, being recorded in only 12.5% and 22.5% of operative notes, respectively.

**Table 3:** Compliance with postoperative management standards before and after implementation of the quality improvement intervention Compliance with postoperative management documentation standards during the baseline audit cycle (October 20-November 19, 2025) and the reaudit cycle (January 20-April 19, 2026) following the implementation of the quality improvement interventions. Comparisons between cycles were performed using Pearson's chi-square test. A p-value of <0.05 was considered statistically significant

Documentation item	Cycle 1 (n = 80) n (%)	Cycle 2 (n = 120) n (%)	χ²	p-value
Postoperative vital signs documented	7 (8.8)	120 (100.0)	173.12	<0.001
Mother ambulation documented	32 (40.0)	120 (100.0)	91.43	<0.001
Feeding plan documented	10 (12.5)	120 (100.0)	155.56	<0.001
Antibiotic prescription documented	37 (46.3)	120 (100.0)	76.44	<0.001
Wound care instructions documented	2 (2.5)	120 (100.0)	190.17	<0.001
Follow-up plan documented	18 (22.5)	120 (100.0)	132.70	<0.001

Following the introduction of the structured operative note template and departmental educational interventions, compliance improved dramatically across all postoperative management domains, reaching 100% for postoperative vital signs, ambulation plans, feeding plans, antibiotic prescriptions, wound care instructions, and follow-up documentation. All improvements were statistically significant (p < 0.001), demonstrating a substantial enhancement in the completeness of postoperative care documentation after implementation of the quality improvement measures.

Domain-level analysis demonstrated substantial improvements across all components of CS operative note documentation following the implementation of the quality improvement interventions (Table 4). During the baseline audit cycle, overall compliance with documentation standards was low, with mean compliance rates of 27.9% for mandatory documentation standards, 18.6% for clinical operative documentation standards, and 22.1% for postoperative management standards.

**Table 4:** Overall compliance with audit standards before and after the quality improvement intervention Domain-level compliance was calculated as the mean percentage compliance across all audit indicators within each domain. Overall compliance represents the average compliance across all audit domains

Audit domain	Cycle 1 mean compliance (%)	Cycle 2 mean compliance (%)	Improvement (%)
Mandatory documentation standards	27.9	99.5	+71.6
Clinical operative documentation standards	18.6	98.2	+79.6
Postoperative management standards	22.1	100.0	+77.9
Overall compliance	22.9	99.2	+76.3

Following the implementation of educational sessions, departmental feedback meetings, visual reminder posters, and a structured operative note proforma, marked improvements were observed across all audit domains. The mean compliance increased to 99.5% for mandatory documentation standards, 98.2% for clinical operative documentation standards, and 100% for postoperative management standards. The greatest absolute improvement was observed within the clinical operative documentation domain, which increased by 79.6 percentage points.

Overall compliance across all audit standards improved from 22.9% during the baseline cycle to 99.2% during the reaudit cycle, representing an absolute improvement of 76.3 percentage points. These findings demonstrate the substantial effectiveness of the implemented quality improvement interventions in enhancing the completeness and quality of CS operative note documentation.

## Discussion

This closed-loop clinical audit demonstrated a substantial improvement in the quality and completeness of CS operative note documentation following the implementation of a multifaceted quality improvement intervention. Baseline compliance across all audit domains was markedly low, particularly for clinical operative documentation and postoperative management records. Following the introduction of a structured operative note proforma, educational sessions, departmental feedback meetings, and visual reminder posters, compliance improved dramatically across nearly all assessed parameters. Overall compliance increased from 22.9% during the first audit cycle to 99.2% during the reaudit cycle, demonstrating the effectiveness of structured quality improvement interventions in improving documentation practices and promoting adherence to established professional standards.

The baseline audit identified significant deficiencies in mandatory documentation standards, including patient identifiers, clinician identification, operative timing, and essential surgical information. These findings are concerning because operative notes constitute a critical component of the patient's clinical and medico-legal record. Accurate operative documentation facilitates continuity of care, supports communication among healthcare professionals, and provides a reliable account of clinical decision-making and surgical management. Professional guidance from the RCOG emphasizes that operative records should be comprehensive, contemporaneous, and sufficiently detailed to support future patient management and medicolegal review [4,7]. Similar deficiencies have been reported in previous audits of CS documentation. Saeed identified substantial omissions in documentation of operative personnel, blood loss estimation, postoperative instructions, and neonatal details, highlighting persistent gaps in obstetric operative note quality despite the availability of documentation standards [2].

One of the most notable findings of the present audit was the extremely poor baseline compliance with documentation of gravidity and parity, gestational age, membrane status, liquor characteristics, assistant surgeon identification, and neonatal parameters. These variables provide important clinical information that may influence postoperative management, future obstetric planning, and interpretation of maternal and neonatal outcomes. Failure to document such information can compromise multidisciplinary communication and hinder effective continuity of care. Similar observations have been reported in previous obstetric documentation audits, where incomplete recording of operative findings, neonatal outcomes, and procedural details represented major deficiencies in surgical recordkeeping [2].

The remarkable improvement observed during the second audit cycle is likely attributable to the introduction of the structured operative note proforma. Standardized documentation templates provide clinicians with a systematic framework that reduces reliance on memory and minimizes omission of essential information. The significant improvements observed across almost all documentation domains in this audit are consistent with previous quality improvement studies evaluating structured operative note systems. Lefter et al. reported that redesigning operative documentation processes significantly improved compliance with documentation standards and reduced variability in operative note quality [8].

Furthermore, the implementation of structured electronic operative note templates has been shown to markedly improve documentation completeness, legibility, and adherence to professional guidelines. Theivendran et al. demonstrated that the introduction of a dedicated electronic operative note template resulted in substantial improvements in documentation quality and achieved full compliance with RCOG standards in several domains [9].

Another important finding was the dramatic improvement in postoperative management documentation. During the baseline cycle, documentation of postoperative vital signs, wound care instructions, feeding plans, follow-up arrangements, and ambulation recommendations was frequently absent. Following the implementation of the intervention package, compliance reached 100% across all postoperative domains. Comprehensive postoperative documentation is essential for ensuring continuity of care, facilitating safe recovery, reducing communication failures, and supporting discharge planning. International guidance on postoperative cesarean care emphasizes the importance of clearly documented postoperative management plans, including nutritional care, early mobilization, postoperative monitoring, thromboprophylaxis, discharge counseling, and recovery planning, to promote patient safety and optimize clinical outcomes [10].

The improvement in neonatal documentation is also noteworthy. Documentation of Apgar scores, birth weight, and neonatal condition increased substantially following intervention implementation. Accurate recording of neonatal outcomes is essential for immediate pediatric management, subsequent clinical review, and institutional quality monitoring. Nicopoullos et al. demonstrated that the introduction of a dedicated CS operative delivery proforma resulted in significant improvements in documentation of neonatal variables and operative findings, with important implications for both clinical governance and medicolegal protection [11].

The success of the intervention likely reflects the combined effect of several complementary quality improvement strategies rather than reliance on a single intervention. Educational sessions increased awareness of documentation standards, departmental meetings promoted engagement and accountability, visual reminder posters provided continuous reinforcement of expected practice, and the structured proforma facilitated compliance during routine clinical work. Multifaceted quality improvement interventions that combine education, feedback, and standardized documentation tools are more effective than isolated interventions because they address both individual and system-level factors influencing clinical practice. Previous audits evaluating structured operative note systems have similarly reported substantial and sustained improvements in documentation quality following the implementation of standardized proformas, audit feedback, and targeted educational measures [8,9].

From a clinical governance perspective, the findings of this audit have important implications. High-quality operative documentation is increasingly recognized as a key indicator of safe surgical practice, effective communication, and professional accountability. Comprehensive records support quality assurance activities, facilitate future audits, strengthen medicolegal protection, and contribute to improved patient safety [8,9]. The near-complete compliance achieved during the second audit cycle demonstrates that substantial improvements can be achieved through relatively low-cost interventions that are feasible even within resource-limited healthcare settings. These findings are particularly relevant for hospitals in low- and middle-income countries, where simple system-level interventions may offer substantial opportunities for improving healthcare quality without requiring major financial investment.

This audit possesses several strengths. The closed-loop design enabled objective assessment of practice change following the implementation of targeted interventions and provided direct evidence of the effectiveness of the quality improvement measures. Inclusion of all consecutive CS operative notes during both audit periods minimized selection bias and provided a representative assessment of routine clinical practice. Furthermore, the audit standards were derived from the internationally recognized RCOG guidance, ensuring that performance was benchmarked against established professional standards [4].

Nevertheless, several limitations should be acknowledged. First, this audit was conducted within a single institution, which may limit the generalizability of the findings to other healthcare settings with different patient populations, documentation systems, or clinical practices. Second, the study evaluated compliance with operative documentation standards rather than maternal or neonatal clinical outcomes; therefore, although documentation quality improved substantially, these findings should not be interpreted as evidence of improved patient outcomes. Third, awareness among healthcare staff that documentation practices were being audited may have contributed to improved performance during the reaudit cycle, introducing the possibility of a Hawthorne effect [12]. In addition, although all reviewers underwent standardized orientation before data collection and used predefined operational definitions, formal assessment of inter-rater reliability was not performed, which may have introduced minor variability in data extraction. Finally, the study did not assess the long-term sustainability or cost-effectiveness of the intervention; therefore, further studies are warranted to determine whether these improvements can be maintained over time and implemented efficiently in other healthcare settings.

Overall, this clinical audit demonstrates that implementation of a structured CS operative note proforma combined with targeted educational and feedback interventions can substantially improve compliance with documentation standards. These findings support the routine use of standardized documentation tools and continuous audit processes as effective strategies for strengthening clinical governance, improving patient safety, and promoting high-quality obstetric practice.

## Conclusions

This closed-loop clinical audit identified significant deficiencies in the quality and completeness of CS operative note documentation. The implementation of a structured operative note proforma combined with educational and feedback interventions resulted in substantial improvements in compliance across all documentation domains. These findings demonstrate that structured quality improvement interventions can effectively enhance the completeness and quality of operative documentation and promote adherence to established documentation standards.

## Appendices

Appendix 1. Structured audit checklist used for data collection

The following structured audit checklist was developed from the Royal College of Obstetricians and Gynaecologists' (RCOG) Good Practice Guidance on Operative Documentation and was used to assess the completeness of cesarean section operative notes during both audit cycles. Each documentation item was evaluated as present (compliant) or absent (noncompliant).

Domain 1. Mandatory Documentation Standards

Documentation Item: Compliance Assessment

Patient name documented: Present/Absent

Patient age documented: Present/Absent

Date of operation documented: Present/Absent

Indication for cesarean section documented: Present/Absent

Primary surgeon documented: Present/Absent

Type of anesthesia documented: Present/Absent

Time of delivery documented: Present/Absent

Clinician name documented: Present/Absent

Date and time of documentation recorded: Present/Absent

Clinician signature documented: Present/Absent

Domain 2. Clinical Operative Documentation Standards

Documentation Item: Compliance Assessment

Gravidity and parity documented: Present/Absent

Gestational age documented: Present/Absent

The assistant surgeon documented: Present/Absent

The anesthetist documented: Present/Absent

Type of abdominal incision documented: Present/Absent

Type of uterine incision documented: Present/Absent

Membrane status documented: Present/Absent

Liquor status documented: Present/Absent

Intraoperative findings documented: Present/Absent

Procedure details documented: Present/Absent

Estimated blood loss documented: Present/Absent

Intraoperative complications documented: Present/Absent

Apgar score documented: Present/Absent

Birth weight documented: Present/Absent

Baby condition at birth documented: Present/Absent

Domain 3. Postoperative Management Standards

Documentation Item: Compliance Assessment

Postoperative vital signs documented: Present/Absent

Mother's ambulation documented: Present/Absent

Feeding plan documented: Present/Absent

Antibiotic prescription documented: Present/Absent

Wound care instructions documented: Present/Absent

Follow-up plan documented: Present/Absent
